# Floquet topological state induced by light-driven band inversion in SnTe

**DOI:** 10.1038/s41567-026-03341-0

**Published:** 2026-06-25

**Authors:** F. Chassot, A. Pulkkinen, G. Kremer, C. Wang, J. Schusser, J. Krempaský, J. Minár, G. Springholz, M. Puppin, J. H. Dil, C. Monney

**Affiliations:** 1 Department of Physics and Fribourg Center for Nanomaterials, Université de Fribourg, Fribourg, Switzerland; 2 New Technologies-Research Center, University of West Bohemia in Pilsen, Plzeň, Czech Republic; 3 Université de Lorraine, CNRS, IJL, Nancy, France; 4 Center for Photon Science, Paul Scherrer Institut, Villigen, Switzerland; 5 Institut für Halbleiter- und Festkörperphysik, Johannes Kepler Universität, Linz, Austria; 6 Lausanne Centre for Ultrafast Science (LACUS), École Polytechnique Fédérale de Lausanne (EPFL), Lausanne, Switzerland

**Keywords:** Topological insulators, Electronic properties and materials

## Abstract

High-intensity coherent light can realize hybrid phases of matter that are not accessible in equilibrium. The coherent interaction between Bloch states and a periodic electric field, which leads to Floquet-engineered band structures, has been extensively discussed in the literature and shown to enable modified electronic states, and it potentially induces topological phases in semiconductors. However, a Floquet topological insulator has not yet been realized experimentally in a semiconductor. Here we show that femtosecond light pulses can create a short-lived topological state in SnTe that is absent from the equilibrium ground state. This occurs when the photo-excitation energy is close to the bandgap of this polar semiconductor. We observe a concomitant renormalization of the band dispersions that reveals the generation of Floquet states connected to the topological state. These results provide a direct experimental observation of a Floquet topological state and indicate that it is driven by a light-induced band inversion in SnTe, although further theoretical work will provide a more detailed characterization of the state. Our work also indicates that optical control of the topological properties of semiconductors on demand is possible.

## Abstract (web summary)

Floquet engineering could, in principle, induce a topological phase in semiconductors, but clear evidence has been lacking. Now it is shown in SnTe that femtosecond light pulses briefly create a topological state that is absent at equilibrium.

## Main

Floquet engineering is a promising route to realize new phases of matter with light and with tailored functionality^1–9^. Time-resolved angle-resolved photoemission spectroscopy (TR-ARPES) directly observes such transient effects with momentum resolution by accessing the out-of-equilibrium electronic structure of the solid in the presence of an intense light field. In 2016, this approach was successfully used to observe Floquet–Bloch states in the semiconductor^10^ Bi_2_Se_3_ and to show that the interaction between the thus created bands led to avoided-crossing gaps^11^. Another intense research effort in recent decades has been the search for materials with new topological properties, which hold promise for applications in spintronics and quantum computing. An open question arising from these two fields of research is whether light could be used to coherently drive a material from a trivial to a topological state, into a phase called a Floquet topological insulator^12,13^. In 2011, a theoretical scheme to generate such a new state of matter in a direct bandgap semiconductor with large spin–orbit coupling was proposed but has not been realized to date^14^.

**Fig. 1:**
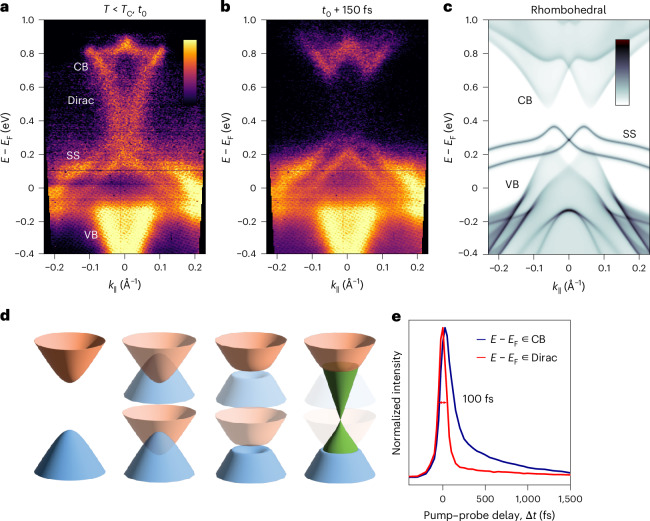
Formation of an ultrashort-lived topological state in polar SnTe through Floquet engineering. **a**, TR-ARPES measurement of SnTe(111) along $\overline{\mathrm{K\varGamma K}}$ using a probe of 6.3 eV and a pump of 0.88 eV, which arrive simultaneously at time ${t}_{0}$. A Dirac cone is observed. **b**, 150 fs after ${t}_{0}$, the Dirac cone has disappeared. **c**, DFT calculations of rhombohedral SnTe(111) with SPRKKR using a semi-infinite stack of layers to calculate the bulk and surface states. In this phase, no Dirac cone is expected at equilibrium, in contrast to the experimental data in **a**. **d**, Schematic illustrations of a Floquet topological insulator, with an initial VB (CB) in blue (orange) that is replicated by the dressing due to the time-periodic electric field of the pump. The replicas interact with the original bands (middle) and, through the hybridization, create the necessary condition for the appearance of a Dirac cone. **e**, Normalized intensity as a function of pump–probe delay integrated over the whole parallel momentum k_|| range and over 0.1 eV centred at +0.85 eV (in the CB) and at +0.45 eV (Dirac cone). The Dirac cone exists in presence of the pump pulse only. SS, surface state. Source data

Here we demonstrate that, by using femtosecond light pulses comparable in energy with the bandgap of the semiconductor SnTe, we are able to transiently modify its electronic structure. As shown in Fig. 1, a new topological Dirac cone, absent in the equilibrium state, appears and exists only as long as the light pulse interacts with the crystal. This occurs via the generation of Floquet states across the bandgap of SnTe and their hybridization with the top of the valence band (VB) and the bottom of the conduction band (CB). On this timescale, the crystal structure is not affected by the photo-excitation, which provides conclusive evidence for the creation of the out-of-equilibrium state of matter known as a Floquet topological insulator. Figure 1d illustrates this process schematically. A resonant and coherent photo-excitation creates a Floquet replica of the VB, which superimposes on the CB, and vice versa. The Floquet replicas hybridize with the original bands, leading to an exchange of band character. The resulting band inversion transforms the equilibrium trivial semiconductor into a non-trivial one. Our experimental results are supported by realistic calculations, including of a semi-infinite crystal within the Floquet formalism.

**Extended Data Fig. 1:**
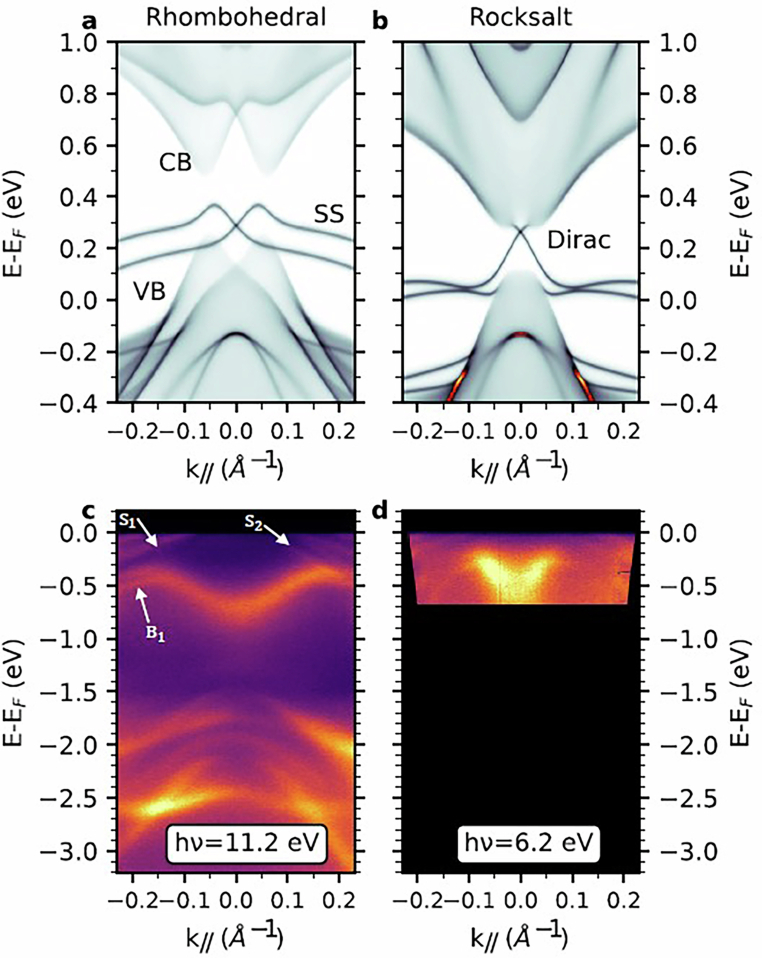
Electronic Structure of SnTe. SPRKKR DFT calculation of the electronic structure of SnTe along $\overline{\mathrm{K\varGamma K}}$ for the rhombohedral (**a**) and rocksalt (**b**) structure to highlight differences: The structural distortion in the polar rhombohedral structure splits the bulk band in panel (**a**), whereas the mirror symmetry in the rocksalt structure protects a topological state in panel (**b**) due to the band inversion; ARPES measurements along the $\overline{\mathrm{K\varGamma K}}$ high symmetry line at 30 K with a photon energy of (**c**) 11.2 eV and (**d**) 6.2 eV with highlighted surface state (${{\rm{S}}}_{1}$), surface resonance state (${{\rm{S}}}_{2}$), and bulk state (${{\rm{B}}}_{1}$); the latter being split because of the break of inversion symmetry in the polar rhombohedral structure.

Notably, the semiconductor SnTe has been investigated during the last decade, because it has been proposed to be a topological crystalline insulator (TCI) protected by mirror symmetries^15,16^. Signatures of linear dispersive states, which are indicative of Dirac cones as predicted by theoretical works, have been found near the Fermi level (*E*_F_)^17,18^. However, a delicate point is that SnTe undergoes a structural transition from a high-temperature rock-salt phase to a low-temperature rhombohedral phase at about 110 K, which is associated with the appearance of a polar, and possibly ferroelectric, state^19,20^. This structural distortion has a direct consequence for the possible existence of a TCI state in SnTe. In the rock-salt phase, a band character inversion between the top of the VB and the bottom of the CB gives rise to the appearance of a Dirac cone at the Γ point of the (111) surface^21^, which is reproduced in our density functional theory (DFT) calculation (Extended Data Fig. 1). By contrast, the low-temperature rhombohedral structural distortion suppresses the band character inversion, thereby eliminating the topological Dirac cone at Γ and leaving only trivial surface states, as depicted in the DFT calculation of Fig. 1c. At the same time, the resulting polar crystal structure leads to a lifting of the band degeneracy of the bulk VB and CB, according to the Rashba model^22^. We previously showed that this Rashba-type spin-splitting and, thus, the structural distortion are persistent up to room temperature, which questions the possible existence of a TCI state in SnTe at any temperature at equilibrium^23^. We here demonstrate a route to create a topological state in SnTe, even in the structurally distorted phase, thanks to Floquet engineering.

## Transient topological state

We start from the rhombohedrally distorted and, thus, topologically trivial state of SnTe. Taking advantage of our previous static ARPES work^23^, we easily identify the $\overline{\mathrm{K\varGamma K}}$ direction in our TR-ARPES experiments at a temperature *T* = 30 K, which is well below the critical temperature *T*_C_ of the transition at 110 K. The short (100 fs) pump pulse of 0.88 eV photo-excites electrons from the VB to the region close to the bottom of the CB. Subsequently, a 6.3-eV probe pulse is used to photoemit electrons from their original or excited states. Figure 1a,b displays TR-ARPES spectra taken at ${t}_{0}$ (when the pump and probe pulse arrive simultaneously) and at ${t}_{0}+150\,\mathrm{fs}$, respectively. A complete measurement as a function of different pump–probe delays is available in the Supplementary Video 1.

**Extended Data Fig. 2:**
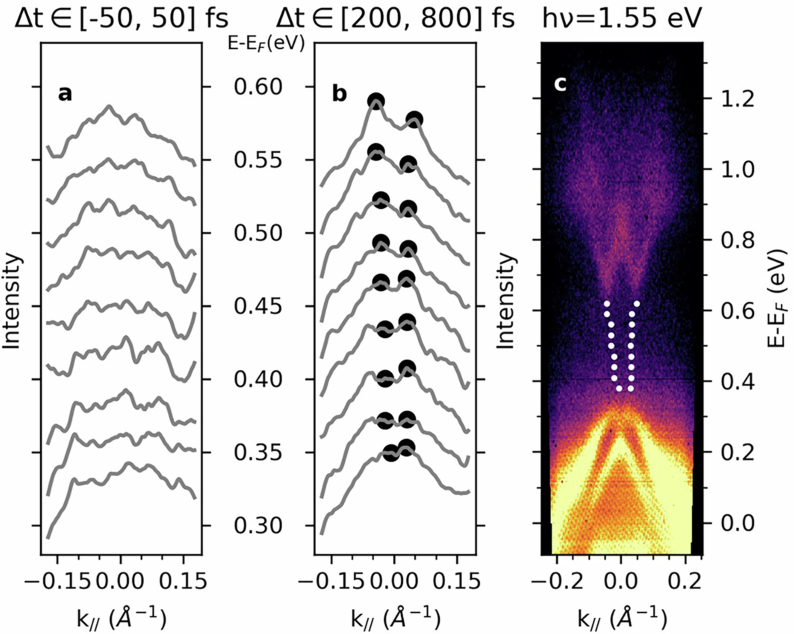
TR-ARPES at 1.55 eV. Momentum distribution curves at their respective energy integrated over 0.1 eV in time range of $\Delta t\in \left[-50,\,50\right]\mathrm{fs}$ (**a**) and $\Delta t\in \left[200,\,800\right]\mathrm{fs}$ (**b**); **c**, TR-ARPES spectra of SnTe(111) using a probe of 6.3 eV and a pump of 1.55 eV, integrated in a time range of 200 and 800 fs. This figure shows that the Dirac cone appears only at longer timescale, indicating a structural transition induced by heat transfer from the pump.

**Extended Data Fig. 3:**
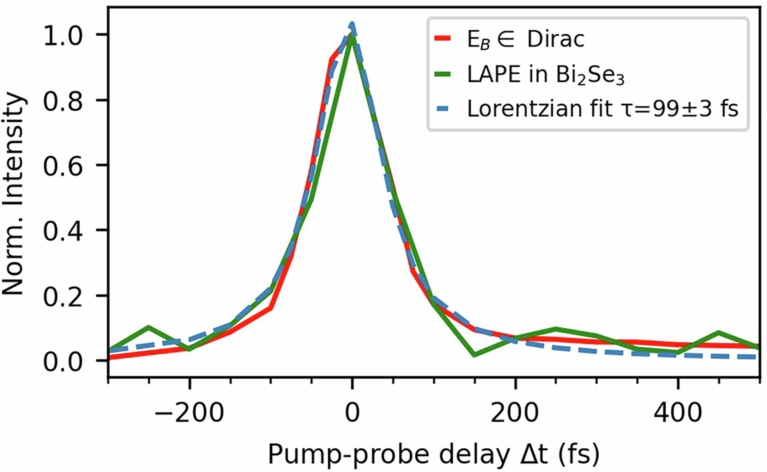
Comparison between the experimental time resolution and the duration of the topological state. Red curve: Normalized intensity of the Dirac cone (integrated in a box over the whole momentum range centered at -0.45 eV for the data pump with 0.88 eV) plotted as a function of pump-probe delays. Green curve: LAPE signal from a reference sample of Bi_2_Se_3_ with the same experimental conditions (the FWHM of the LAPE signal is known in the literature to depend solely on the pump-probe cross correlation) and a Lorentzian fit of the signal of the Dirac cone in blue.

Several bands are visible in the data at ${t}_{0}$, including a trivial surface state, a bulk VB and the transiently populated CB (the corresponding electronic structure is given on a larger scale in Extended Data Fig. 1). However, the most striking feature is that, despite being in the rhombohedral phase, a Dirac cone crosses the bandgap at a similar position as the topological Dirac cone in the rock-salt structure (Supplementary Discussion and Extended Data Fig. 2). At longer timescales, after the photo-excitation pulse is gone, the Dirac cone disappears and the CB exhibits the W shape predicted by DFT (Fig. 1c). The time evolution of the transient Dirac cone shown in Fig. 1e clearly displays different behaviour compared with the electrons excited to the bottom of the CB. Although these electrons remain in the CB for several hundred femtoseconds, the transient Dirac cone has a duration of about 100 fs only. This is very unusual, as electrons in many topological systems typically remain in the Dirac cone for much longer due to a bottleneck^24–26^. This sharp and symmetrical intensity profile is, therefore, as short as it can be, as it is limited purely by our experimental time resolution (see Extended Data Fig. 3 for a measurement of our time resolution). The electronic structure giving rise to the Dirac cone exists in the presence of the photo-excitation pulse only.

## Not a structural transition

**Extended Data Fig. 4:**
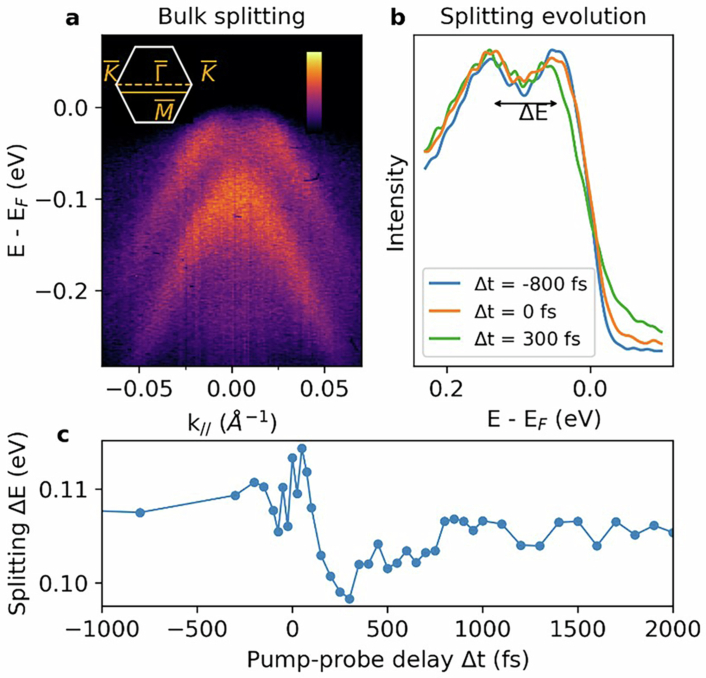
Evolution upon pumping of the bulk Rashba splitting as a function of pump-probe delay to prove the absence of a structural transition at t0. **a**, ARPES measurement taken along $\overline{\mathrm{K\varGamma K}}$ but shifted by ${k}_{//}=0.13\,{\mathop{{\rm{A}}}\limits^{\circ }}^{-1}$ towards $\overline{{\rm{M}}}$ (see continuous line in the Brillouin zone) where the bulk band is split because of the rhombohedral distortion; **b**, EDCs as a function of pump-probe delay measured at$\,{k}_{//}\in [-0.03,\,0.03]\,{\mathop{{\rm{A}}}\limits^{\circ }}^{-1}$; **c**, Time evolution of the bulk splitting extracted from fits to the EDCs.

The ultrashort duration of the Dirac cone indicates that its origin is not due to a structural change, which typically occurs on a longer timescale^27–29^. In our experiment, we could actually access the time evolution of the structural distortion through the Rashba splitting of the bulk bands, and we did not see any substantial change within the first 150 fs after ${t}_{0}$, which proves the absence of notable atomic displacement (Extended Data Fig. 4). An estimation of the transient temperature increase at the highest fluence used here yields 34 K (Supplementary Discussion). This further confirms the coexistence of the rhombohedral distortion with a topological state induced by an intense ultrashort light pulse at ${t}_{0}$.

## Floquet renormalization

**Fig. 2:**
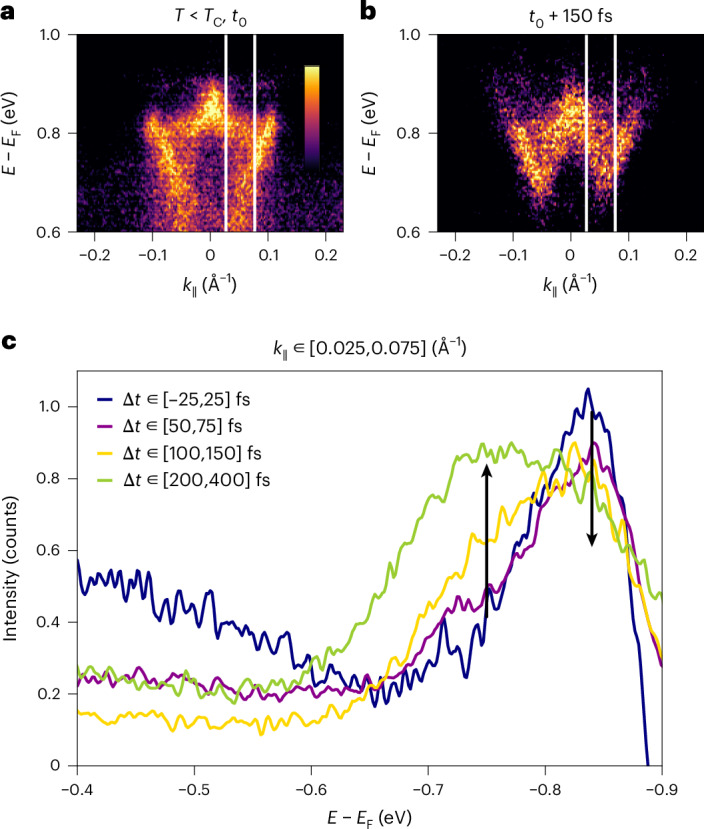
Floquet renormalization of the CB. **a**, TR-ARPES spectrum of SnTe(111) using a pump of 0.88 eV and integrated between pump–probe delays of −25 fs and 50 fs. **b**, Same integrated over 100 fs to 200 fs. **c**, Energy distribution curves for different pump–probe delays integrated between the two white lines in **a** and **b**. The flat band that appears at ${t}_{0}$ is due to Floquet states. It is later replaced by the original W-shaped CB. The black arrows indicate their evolution in time. Source data

**Extended Data Fig. 5:**
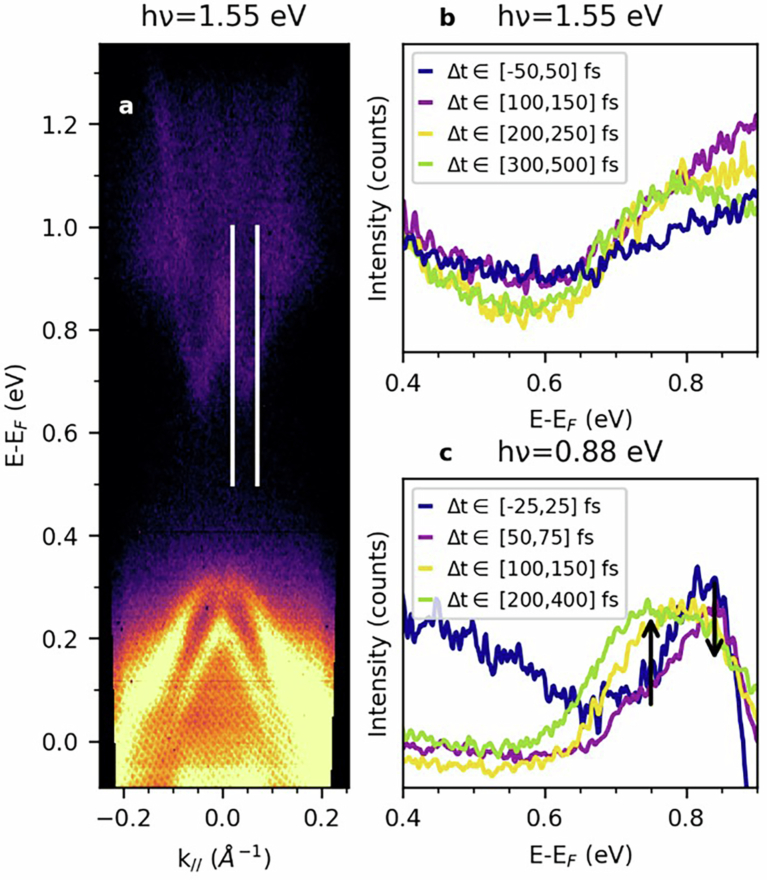
Change of the conduction band dispersion. **a**, TR-ARPES spectra of SnTe(111) using a probe of 6.3 eV and a pump of 1.55 eV, integrated between -25 and 800 fs of pump-probe delays ; **b**, EDCs for different pump-probe delays for a pump photon energy of 1.55 eV integrated in momentum between the two white lines in panel (**a**) showing no band renormalization but only relaxation of electrons from the top to the bottom of the CB; **c**, same as in (**b**) but for a pump photon energy of 0.88 eV indicating the renormalization of the CB.

**Extended Data Fig. 6:**
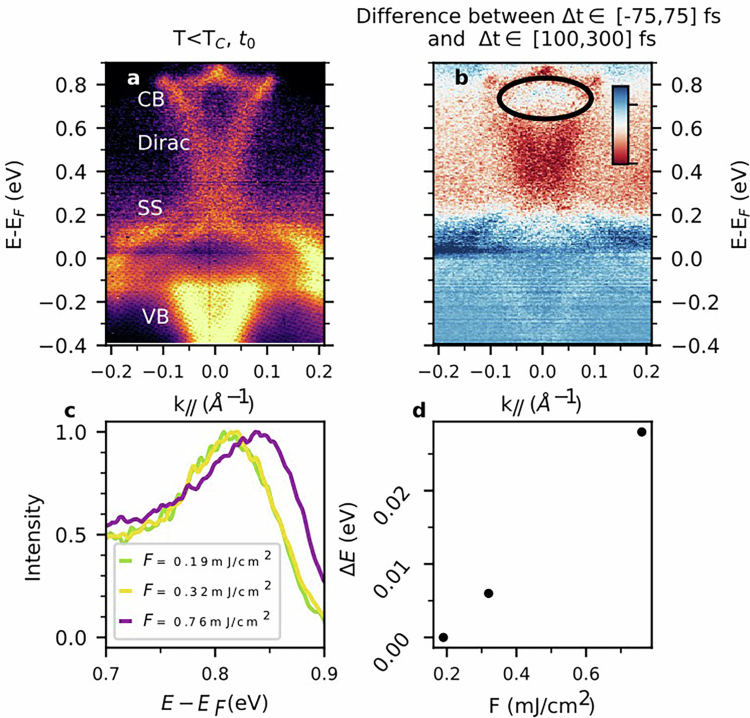
Renormalization of the Conduction Band. **a**, TR-ARPES measurement of polar SnTe(111) using a probe of 6.3 eV and a pump of 0.88 eV that arrive simultaneously on the sample along $\overline{\mathrm{K\varGamma K}}\,$; **b**, Difference between the TR-ARPES measurement integrated between [-75, 75] fs and [100, 300] fs (red: more signal at t_0_; blue more signal after t_0_). The difference spectrum indicates a change in the conduction band (highlighted in black) going from a flat to a ‘W’ shape; **c**, Selection of EDCs extracted at ${t}_{0}$ and integrated between ${k}_{//}\in [-0.05,\,0.05]\,{\mathop{{\rm{A}}}\limits^{\circ }}^{-1}$ for a photoexcitation of 0.88 eV photon energy and varying fluences; **d**, extracted shift of the hybridized CB from panel (**c**) showing a linear trend.

Concomitantly to the appearance of the Dirac cone at ${t}_{0}$, we observe that the CB displays an anomalous flat dispersion. In Fig. 2, a zoom-in of our ARPES data for the transiently occupied CB is shown near ${t}_{0}$ and compared with later times. It can be seen that the photoemission intensity is particularly pronounced along a flat band at 0.8 eV above *E*_F_ during the first 100 fs, and then at later times, it is distributed more towards lower binding energies, emphasizing the W-shaped CB bottom, as expected from DFT (Fig. 1c). We characterize the duration of this effect by plotting energy distribution curves across the side of the CB at different time delays, which yields two further hints on what is happening. First, at around 0.8 eV above ${E}_{{\rm{F}}}$, the energy distribution curve can be decomposed into two peaks, rather than being a continuous electron distribution that changes shape as a function of time, as would be expected for an excited population of electrons in a single band and as is observed for an excitation energy of 1.55 eV (Extended Data Fig. 5). Second, the upper peak (near 0.85 eV above ${E}_{{\rm{F}}}$), which comes from the flat CB, exists only for very short time delay, up to 100 fs, as also visible from the difference plot (Extended Data Fig. 6). Altogether, we observe that the transient flat band appears during photo-excitation only, indicating that a renormalization of the CB dispersion occurred due to hybridization with Floquet–Bloch sidebands.

## Fluence and photon energy dependence

**Fig. 3:**
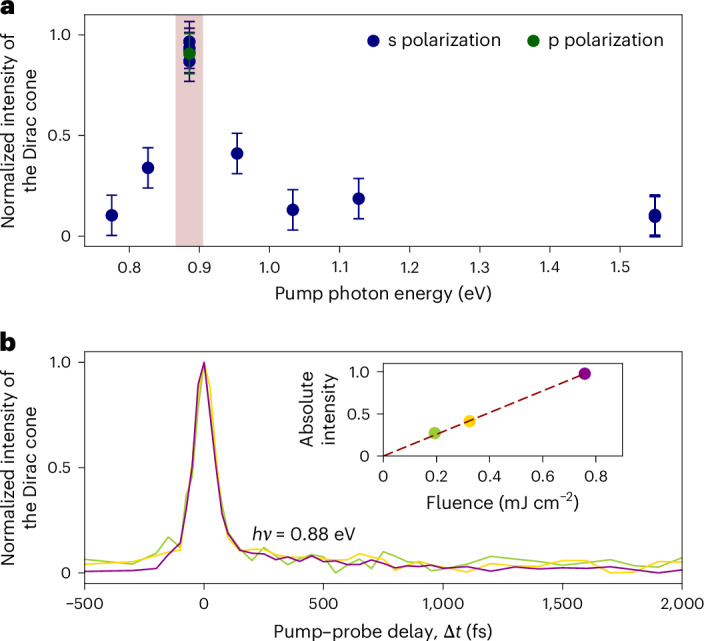
Investigation of the Dirac cone as a function of fluence and pump photon energy. **a**, Evolution of the intensity of the Dirac cone as a function of pump photon energy. To normalize by the amount of absorbed energy, the maximum of the signal of the Dirac cone (integrated over *E* − *E*_F_ $\in \left[0.35,\,0.45\right]$ eV and$\,{k}_{\parallel }\in [-0.03,\,0.03]\,{\mathop{{\rm{A}}}\limits^{\circ }}^{-1}$) is divided by the maximum of the signal from the surface state. A resonance was observed near the energy of the bandgap at the $\bar{\Gamma }$ point. The error bars were estimated based on contamination due to inelastic secondary electrons photo-excited in the bandgap. **b**, Normalized intensity as a function of pump–probe delay for different fluences for a signal integrated over the same region in **a** for a pump energy of 0.88 eV. Inset: absolute intensity of the Dirac cone as a function of fluence for the same parameters. No finite lifetime was observed. Source data

**Extended Data Fig. 7:**
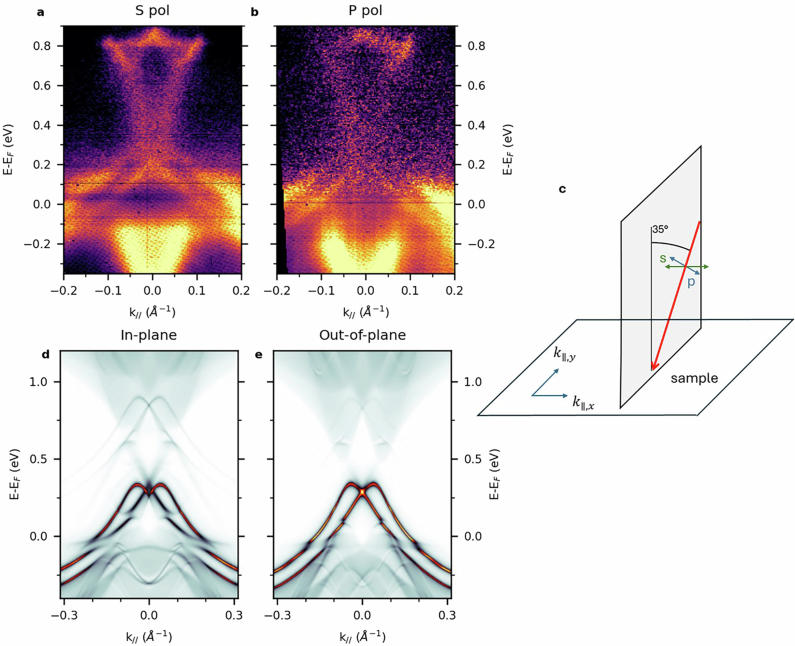
Comparison between Dirac cones with s- and p-polarized photoexcitation at 0.88 eV photon energy. TR-ARPES measurement integrated between [-75, 75] fs for **a** s-polarized and **b** p-polarized photoexcitation. Electronic structure calculations for a semi-infinite crystal within the Floquet formalism show that a purely **a** in-plane light polarization has a stronger effect than **b** a purely out-of-plane polarization; **e**, the geometry of our experiment (with an incident angle of 35°) indicates that the effect of p-polarization photoexcitation in principle be reduced to 82% of our s-polarization data, due to the reduced in-plane component of the pump electric field in our experimental geometry.

**Extended Data Fig. 8:**
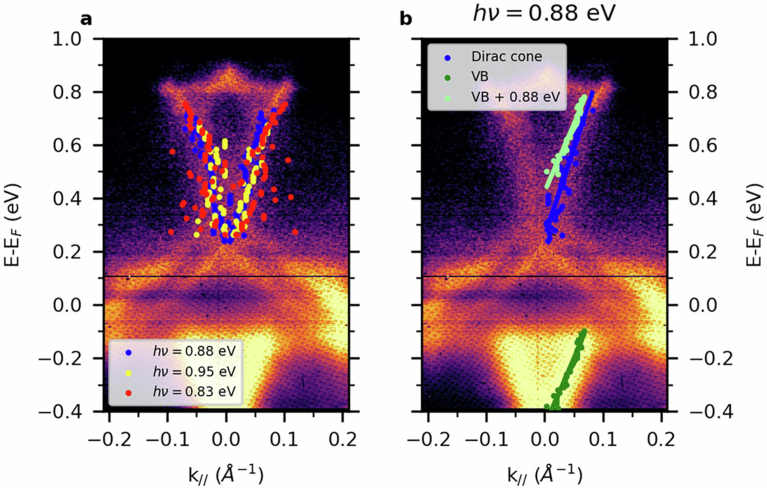
Comparison between Dirac cones at different photon energies and VB. TR-ARPES spectra of SnTe(111) obtained using a probe of 6.3 eV arriving synchronously with a pump of 0.88 eV, where on top of that in panel (**a**) we display the maxima extracted from MDC upon pumping with 0.88 eV (blue dots) and, slightly off-resonance at 0.95 eV (yellow) or 0.83 eV (red); (**b**) same than panel (**a**) for a comparison between the Dirac cone obtained with a 0.88 eV pump photon energy and the VB (dark green), shifted by the same pump photon energy (light green), highlighting the different dispersions.

Having shown that a Dirac cone can be photo-induced at ${t}_{0}$ by an ultrashort light pulse, we now provide evidence that this effect occurs only close to an optical resonance between the extrema of the bulk VB and CB. Figure 3a shows the normalized intensity of the Dirac cone versus the pump photon energy, which reveals several key points. First, using s and p linear polarizations for the pump photon yields, in both cases, a Dirac cone, which rules out laser-assisted photoemission as the dominant effect^12,30^ (see Extended Data Fig. 7 for the TR-ARPES spectra and the experimental geometry). Second, the pronounced maximum of the effect occurs at a photo-excitation energy of 0.88 eV, resonant with the transition near the extrema of the VB to the CB at the $\bar{\Gamma }$ point. The cone appears at the same energy relative to *E*_F_ for photo-excitations of 0.83 eV and 0.95 eV, which also excludes the possibility of a simple replica, whether laser-assisted photoemission or Floquet (Extended Data Fig. 8a). This is also confirmed because the Dirac cone appearing at those pump photon energies does not display the same dispersion as the initial VB (Extended Data Fig. 8b). These points provide solid evidence for a Floquet-induced topological state, where maximal hybridization occurs when the replicas and original bands overlap.

The fluence dependency at a pump photon energy of 0.88 eV, shown in Fig. 3b, indicates that the lifetime of the Dirac cone remains unaffected by the fluence. That is, it is independent of the deposited energy or the total excited electron population. Furthermore, the inset of Fig. 3b shows that the evolution of the absolute intensity as a function of fluence displays a clear linear behaviour. In addition, the small displacement of the upper peak of the flat CB (Fig. 2c), which is due to the hybridization gap with the Floquet replica of the VB, also follows a linear trend as a function of fluence (Extended Data Fig. 6). These linearities indicate that the effect scales quadratically with the incoming electric field and converges to zero without photo-excitation. It is, thus, reminiscent of the optical Stark effect as a consequence of the generation of Floquet states driven by the optical field of the photo-excitation^31^.

## Discussion and outlook

**Fig. 4:**
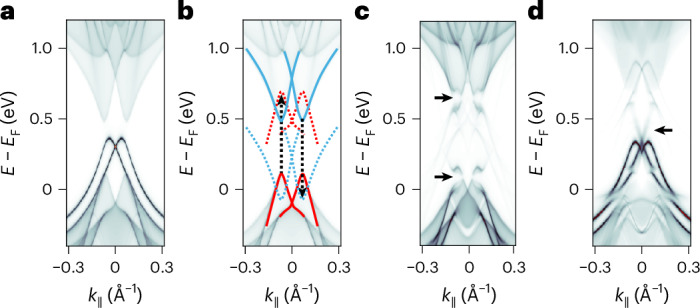
Calculation of the electronic structure of distorted SnTe(111) within the Floquet formalism. **a**, Ground state from the semi-infinite DFT calculations, showing the Rashba-split surface (sharp bands at 0.3 eV above *E*_F_) and the bulk states. **b**, Like **a** but excluding the surface contribution and with a guide to the eyes for the VB (red) and CB (blue). The corresponding Floquet replica (dashed lines) is shifted by the pump photon energy. **c**, Bulk-projected electronic structure within the Floquet formalism for an electric field strength of 0.3 GV m^−1^. Replicas of the CB, VB and hybridization gaps with the original bands (black arrows) are visible. **d**, As in **c** but showing only the surface contributions. The Dirac cone appears between the hybridized bands (black arrow). Source data

**Extended Data Fig. 9:**
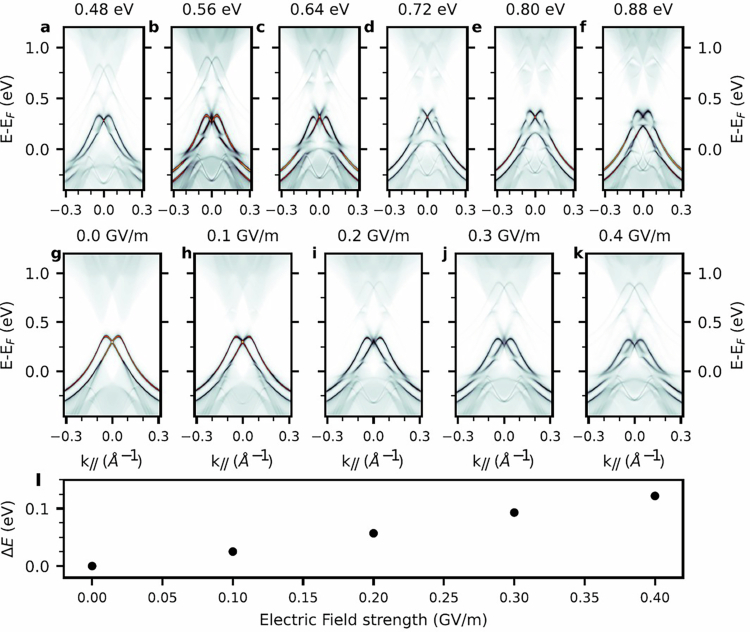
Calculation as a function of pump photon energy and electric field strength. **a – f**, Calculation of the bulk and surface electronic states within the Floquet formalism with a varying pump photon energy and an electric field strength kept constant at 0.3 GV/m; **g – k**, Calculation of the bulk and surface electronic states within the Floquet formalism with a pump photon energy of 0.56 eV and a varying electric field strength; **l**, Displacement of the CB after hybridization, showing a linear trend.

To support our experimental observations, we performed DFT calculations using a semi-infinite crystal within the Floquet theory. Figure 4a shows the electronic structure near the Γ point without any light electric field. We simulated our experimental set-up by doing calculations for a pump photon energy of 0.56 eV to account for the smaller bandgap than in the experiment. With this photon energy, the Floquet replica of the VB is expected to overlap on the CB, and vice versa (see the guide to the eyes in Fig. 4b). The next two panels display our main theoretical results. The bulk and surface electronic band structure of SnTe from Floquet theory are shown in Fig. 4c,d, respectively. In the presence of the electric field of the pump pulse, several crucial effects can be observed. (1) Hybridization gaps open in the bulk VB and bulk CB at about 0.1 eV and 0.7 eV above *E*_F_, respectively (black arrows in Fig. 4c). The flat CB in Fig. 2 is their consequence. (2) A cone-like surface state appears within the bandgap (black arrow in Fig. 4d), which connects the flat CB to the trivial surface state. This is the Dirac cone observed in Fig. 1a. (3) These effects are most obvious when the Floquet replica of the bulk VB and CB overlap with the original bands, such that hybridization gaps occur near the extrema of the bulk bands (Extended Data Fig. 9). This explains the resonant behaviour illustrated in Fig. 3. (4) Finally, the hybridization gaps and the Dirac cone are stronger for an s-polarized than for a p-polarized light electric field, in agreement with our experimental data (Extended Data Fig. 7).

**Extended Data Fig. 10:**
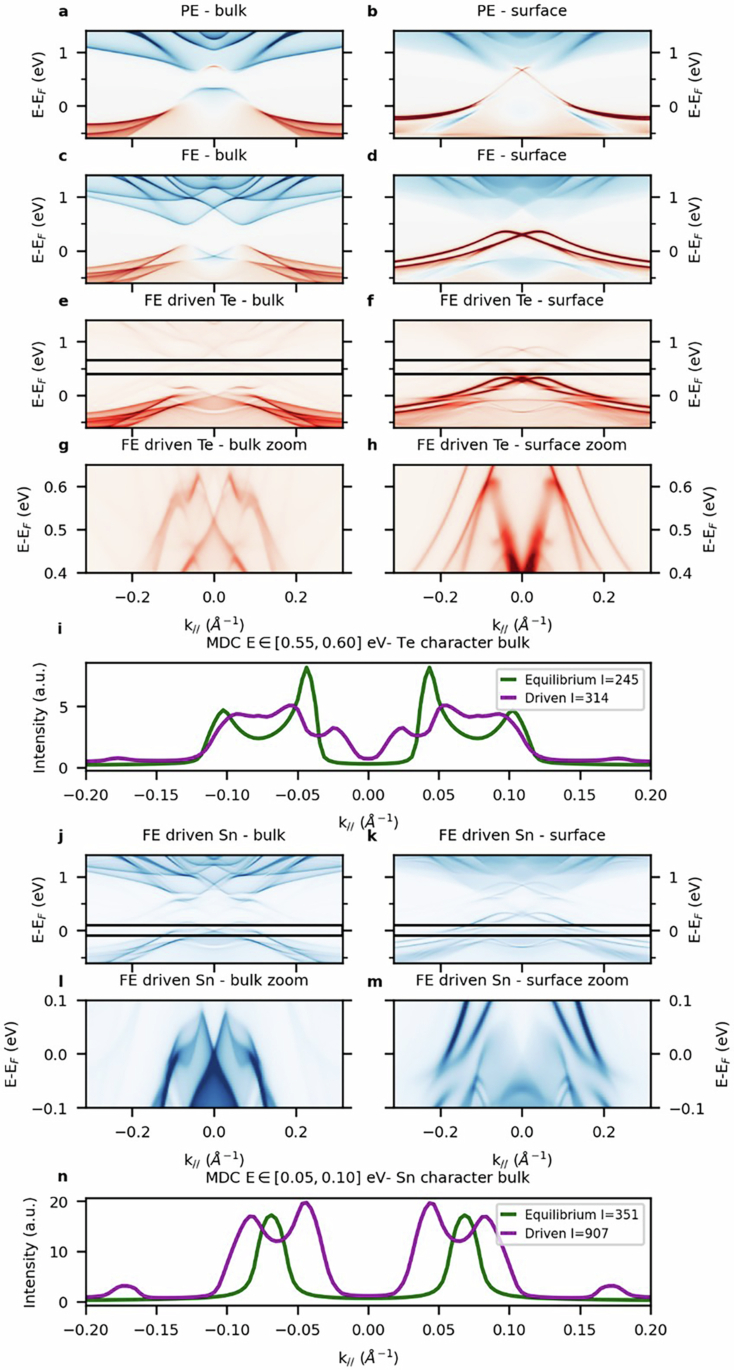
Orbital characters in Wannier-projected Bloch spectral function: band inversion. **a – b**, Difference plot between Te (red) and Sn (blue) contribution in rocksalt SnTe, for bulk and surface respectively, in agreement with literature (ref. ^21^); **c – d**, same for the rhombohedral state at equilibrium; **e – f**, Te contribution for the rhombohedral states driven by a pump energy of 0.56 eV and electric field strength of 0.3 GV/m; **g – h**, zoom of (**e** – **f**) at the hybridization band gap; **i**, MDC of the Te contribution in the driven and at equilibrium state integrated between [0.55, 0.60] eV above the Fermi level showing an increase of the Te character close to the hybridization gap at the CB (the legend indicates the total intensity integrated for both cases). **j – n**, Similar analysis applied for the VB area and the Sn character.

Therefore, both our calculations and experimental observations strongly indicate that the ultrashort-lived topological state appears transiently at ${t}_{0}$ because the hybridization gaps open (Fig. 4c) due to the Floquet physics. Small changes in the orbital character of the bands near the hybridization gaps indicate that a band inversion is driven by the pump light field (Extended Data Fig. 10). Note that their magnitude increases linearly with increasing electric field (Extended Data Fig. 9), as expected for an optical Stark effect (Extended Data Fig. 6). Moreover, the peak field strength used in the calculations for Fig. 4 is in excellent quantitative agreement with our experimental value ($3\,\times \,{10}^{8}$ V m^−1^). We propose that the exchange of orbital character between the top of the VB and the bottom of the CB created by Floquet hybridization gaps is key to the appearance of the Dirac cone. This bears some similarity with the band structure predicted for the rock-salt structure that leads to a TCI phase at equilibrium^20^. This, in turn, alters the topological invariant of SnTe, as it allows for the transient appearance of the Dirac cone at the Γ point, as long as the Floquet states exist.

A striking result is the coexistence between maximally Rashba-split bands and a Dirac cone that appears in the presence of the photo-excitation only. The former is due to the rhombohedrally distorted lattice structure, which breaks inversion symmetry in the bulk. This is fundamentally different from the situation at equilibrium, for which a Dirac cone is not allowed in the presence of a large lattice distortion and would typically be present for a rock-salt structure^21^. This is the reason why no Dirac cone is observed after 150 fs in the absence of light, despite the relatively long occupation of the CB bottom (about 1 ps; Fig. 1e). This means that we have generated with light a new out-of-equilibrium state of matter, for which a topological state exists in the presence of inversion symmetry breaking in SnTe. This is the essence of the Floquet topological insulator state.

These results raise the question of why such physics can be realized in the polar semiconductor SnTe but has not been observed in any other system despite substantial efforts. This is especially the case because, in comparison with other examples of Floquet physics^1,7–10,32,33^, for which high photo-excitation fluences at low photon energies have been used, the experimental conditions here, involving relatively mild fluences and high photon energies, might be surprising. The resonance condition of the near-bandgap photo-excitation, which is at the origin of the optical Stark effect, enhances such an effect at low fluence, as evidenced by the linear dependency on the fluence^31^ (Fig. 3b). Moreover, we speculate that the polar nature of SnTe makes it particularly responsive to the dipolar field of light. Finally, although SnTe is not in a TCI phase at low temperature, it is very close to a topological phase transition and, therefore, highly susceptible to a light-induced transformation. The exact topological nature of this transient phase remains to be determined, and we hope that our work will trigger new theoretical frameworks for its identification.

## Methods

### Sample growth

Epitaxial SnTe(111) films of thickness 2 μm were grown by molecular beam epitaxy on BaF_2_ substrates under ultrahigh-vacuum conditions at a substrate temperature of 350 °C in a compound effusion cell. During growth, the SnTe(111) surface exhibited a perfect two-dimensional reflection high-energy electron diffraction pattern, thus revealing a perfect two-dimensional growth mode. After growth, the samples were transferred to the ARPES set-up without breaking the ultrahigh-vacuum conditions using a battery-operated vacuum suitcase having a pressure of lower than ${10}^{-10}$ mbar. Note that due to the high density of native Sn vacancies, SnTe intrinsically exhibits a high p-type carrier concentration of typically $2\times {10}^{20}\,{\mathrm{cm}}^{-3}$. For this reason, the Fermi level is always inside the topmost VB. At room temperature, the lattice parameter of the SnTe layers was determined to be *a* = 6.325 Å (rhombohedral lattice parameter of 4.472 Å), which is in good agreement with literature values^18^. In our previous paper, we characterized the SnTe epilayer with high-resolution X-ray diffraction to prove the perfect (111) orientation of the lattice and the high quality of our samples^22,34^.

### (TR-)ARPES measurements

The TR-ARPES investigation were carried out using a Scienta DA30 photoelectron analyser with a base pressure lower than $3\,\times \,{10}^{-11}$ mbar. The photon sources used for TR-ARPES were based on a femtosecond laser (Pharos, Light Conversion) operating at 1,030 nm. Half of its power was converted into 780-nm light with an optical parametric amplifier, which was then frequency-quadrupled to 6.3 eV in β-BaB_2_O_4_ crystals to generate probe pulses^35^. The remaining half of the fundamental laser power was directed into a collinear optical parametric amplifier (Orpheus, Light Conversion) to generate pump pulses between 800 nm and 1,600 nm (1.55–0.77 eV) with a duration of about 50 fs. The total temporal resolution was estimated to be equal or better than 100 fs by measuring the width of the cross-correlation between the pump and the probe pulses and using a laser-assisted photoemission signal on a reference sample of Bi_2_Se_3_. We used s- and p-polarization for the pump and an incident angle of 35° with respect to the surface normal and acquired the data with a negative bias of −5 V. The total energy resolution was about 30 meV, and the angular resolution was 0.1°. The samples were cooled to 30 K at rates <5 K min^−1^ to avoid thermal stress.

Incident fluence values are given in the main text. The corresponding peak electric field was about $3\,\times \,{10}^{8}$ V m^−1^.

### Calculations

The fully relativistic DFT calculations of the SnTe(111) surface were performed with the screened Korringa–Kohn–Rostoker method in the SPRKKR package^36^. The Te-terminated SnTe(111) surface was modelled as a semi-infinite stack of layers in the atomic sphere approximation, with the basis set truncated at lmax = 2. Exchange-correlation effects were treated at the level of the local spin density approximation using the parameterization of Vosko, Wilk and Nusair^37^. The lattice parameters in the hexagonal basis were *a* = 4.528 Å and *c* = 11.090 Å for the rock-salt structure and *a* = 4.523 Å, *c* = 11.227 Å and *z*_Te_ = 0.483 for the rhombohedral structure. The surface-projected electronic band structures, showing kz-integrated bulk bands and the surface states of the SnTe(111) surfaces, are represented by the Bloch spectral function.

The SnTe bulk electronic structure, including the pump photon field, was calculated using the projector augmented-wave method (Quantum Espresso^38,39^ v7.0) with a 9 × 9 × 9 *k*-point grid and the Perdew–Burke–Ernzerhof exchange-correlation functional^40^. Tight-binding parameters for the *s* and *p* orbitals of Sn and Te were obtained using Wannier90^41^. The spin–orbit interaction was included in all calculations.

The coupling of the pump photon field to the SnTe(111) surface was included through the Floquet formalism. The time-dependent Hamiltonian $H\left(t\right)={H}_{0}\,\exp [{\rm{i}}(e/\hslash ){\bf{A}}(t)\cdot {\bf{R}}]$ was expanded using the Jacobi–Anger identity, resulting in coupling between the photon-dressed band replicas. This yielded a block-structured extended Hamiltonian, which we truncated at the first-order Floquet sidebands. To study the surface properties, the bulk Hamiltonian was reorganized into hopping matrices describing intralayer (${H}_{00}$) and interlayer (${H}_{01}$) interactions. The semi-infinite surface Green’s function was obtained using the iterative method^42^ of López Sancho et al., which we extended to operate in the photon-dressed (Floquet) Hilbert space by applying the iteration to the extended layer Hamiltonians.

## Online content

Any methods, additional references, Nature Portfolio reporting summaries, source data, extended data, supplementary information, acknowledgements, peer review information; details of author contributions and competing interests; and statements of data and code availability are available at 10.1038/s41567-026-03341-0.

## Supplementary information

Supplementary Information Supplementary Methods and Supplementary Discussion.

Supplementary Video 1 TR-ARPES spectra of SnTe(111) using a probe of 6.3 eV and a pump of 0.88 eV for different pump–probe delays.

## Source data

Source Data Fig. 1 Electronic structure data.

Source Data Fig. 2 Electronic structure data.

Source Data Fig. 3 Unprocessed energy and delay dependant data.

Source Data Fig. 4 Unprocessed calculation data.

## Data Availability

The experimental data are available via OLOS, a public repository, at 10.34914/olos:2dba2ewtjjblbildchgwh5n3ty. Any additional information can be acquired from the authors upon reasonable request. Source data are provided with this paper.

## Code availability

The SPRKKR multiple scattering package is freely available (no costs apply) under the specific user licence. The package can be downloaded following registration at https://sprkkr.org. The Python-based interface, ase2sprkr, is published under the MIT licence at https://github.com/ase2sprkkr/. The tight-binding code used to calculate semi-infinite surface Floquet states is available upon reasonable request from the corresponding authors. All input files, postprocessing procedures and scripts used to process the theoretical data are available from the authors on request.
