# Supplementary material for: Floquet topological state induced by light-driven band inversion in SnTe
Source: Nat Phys. 2026 Jun 25;22(8):1282–6. doi: 10.1038/s41567-026-03341-0 (PMC13461349; doi:10.1038/s41567-026-03341-0)
Supplement: Supplementary file 1 [file 41567_2026_3341_MOESM1_ESM.pdf]

nature physics

Supplementary information

https://doi.org/10.1038/s41567-026-03341-0

Floquet topological state induced by light-driven band inversion in SnTe

In the format provided by the authors and unedited

T

Table of Contents – supplementary information

Supplementary Information 2
Supplementary Methods 2
Normalization procedure 2
Supplementary Discussion 2
TR-ARPES data with 1.55 eV pump photons and temperature increase 2
Orbital characters in Wannier-projected Bloch spectral function: band inversion 3
Supplementary video 3

Supplementary Information

Supplementary Methods

Normalization procedure

In this paper, we employ various normalization procedures tailored to the specific comparisons or highlights we aim to achieve. For the time-resolved band structures (e.g., Fig. 1a-1b), each momentum distribution curve (MDC) is divided by its maximum value. This enables us to simultaneously plot both the valence band (VB) and conduction band (CB), despite the differing signal intensities.

In Figures 1e and 3b, the intensity integrated within a defined region is normalized by its maximum value to compare the lifetimes of different states. For the inset of Figure 3B, we plot the maximum absolute intensity of the Dirac cone signal, subtracting the signal before t_0 for background removal.

In Figure 3a, to compare the occupation of the topological state under varying fluences and wavelengths, we normalize the maximum intensity of the Dirac cone by the maximum intensity of the surface states above the Fermi level (integrated over $\mathrm{E} \in [0.15, 0.25]$ eV and $k_{//} \in [-0.2, 0.2]$ Å^{-1}). This signal serves as the closest approximation to the amount of absorbed energy.

Supplementary Discussion

TR-ARPES data with 1.55 eV pump photons and temperature increase

Extended Data Fig. 2 shows the TR-ARPES spectra of SnTe(111) measured at 30 K pump with a photon energy of 1.55 eV with an incident fluence of 0.29 $\mathrm{mJ/cm^2}$. The data at t_0 shows no Dirac cone, indicating that we do not induce a Floquet topological insulator in that case. It is however worth noting that, at longer timescale, we observe indication of a Dirac cone in the EDCs. Because of the temporal delay between t_0 and the appearance of the effect, we attribute this Dirac cone to a topological transition induced by heat transfer induced by the pump.

To estimate the change of temperature, we perform the following estimation: as the specific heat c_L depends on the temperature T, we use the following formula $c_L(T) = c_{L,300} + c_1 \frac{\left(\frac{T}{300K}\right)^{\alpha} - 1}{\left(\frac{T}{300K}\right)^{\alpha} + \frac{c_1}{c_{L,300}}}$, where $c_{L,300}$ is the specific heat at 300 K and $c_1 = 115$ J/kg K and $\alpha = 0.63$ are material properties. Furthermore, from the work of Suzuki and Adachi[43], we elaborate that the penetration depth of SnTe is $\lambda(h\nu = 1.55 \text{ eV}) = 15$ nm. As we have a reflectivity of $R = 0.65$ at this wavelength, a fluence $F = 0.29 \ \mathrm{mJ/cm^2}$, a mass density[44] of $\rho = 6.445 \ \mathrm{g/cm^3}$, we can therefore solve the following equation

$$\int_{T_I}^{T_F} C_L(T)dT = \frac{(1-R)F}{\rho\,\lambda}$$

Starting from $T_I = 30$ K therefore yields $T_F \cong 116$ K, so that the critical temperature T_C of the second-order phase transition is reached.

This agrees with the reduction of the Rashba splitting at the same timescale measured with every pump photon energy and is in agreement with previous literature. In the specific case of Extended Data Fig. 4 using a pump energy of 0.88 eV and a fluence of $F = 0.75 \ \mathrm{mJ/cm^2}$, we can iterate the same strategy than above to estimate the temperature increase : first, we adapt the penetration depth at this photon energy $\lambda(h\nu = 0.88 \text{ eV}) = 134$ nm and the corresponding reflectivity $R = 0.45$. Then, by solving the same equation and starting from $T_I = 30$ K, result

yields $T_F \cong 64$ K, lower than $T_C = 110$ K and consistent with the decrease of splitting by 10% depicted in Extended Data Fig. 4.

<u>Orbital characters in Wannier-projected Bloch spectral function: band inversion.</u>

In Extended data Figure 10, we further analyze our calculations for the rocksalt structure (without pump), rhombohedral structure (without pump) and rhombohedral structure with a pump energy of 0.56 eV and electric field strength of 0.3 GV/m. By distinguishing the Wannier-projected Bloch spectral functions as a function of their center locations (Te vs Sn), we can show the difference between Te (red) and Sn (blue) contributions in each case, be it for the bulk (left part) or the surface states (right part). In the rocksalt state (panel a – b), we therefore reproduce the result of Plekhanov and coworkers (see reference 21), by showing a clear inversion of band character between the VB and CB. In the rhombohedral undriven state (panel c – d), we also show that the CB is of Sn character only, while the VB is mostly of Te character.

Turning now our attention to the rhombohedral structure driven by the pump described above, the situation requires a deeper analysis: in panel e – f (j – k) we show the Te (respectively Sn) contribution. For the case of a light-induced band inversion, we expect a change of character of the original bands close to their overlap with the Floquet replica, and therefore show a zoom at the bottom of the CB (panel g – h) and top of the VB (panel l – m). Upon the extraction of momentum distribution curve at the respective bottom of the CB for the Te case (panel i) and top of VB for the Sn case (panel n), we can compare the intensity at equilibrium and for the driven state. The intensity is increasing upon photoexcitation, which is an indication of a change of the orbital character near the hybridization gap.

Supplementary Video

TR-ARPES spectra of SnTe(111) using a probe of 6.3 eV and a pump of 0.88 eV for different pump-probe delays.
